# Do brief mindfulness-based interventions improve sport-related performance? A systematic review

**DOI:** 10.3389/fpubh.2026.1702327

**Published:** 2026-02-05

**Authors:** Shudian Cao, Yuantao Cheng, Jia Liu, Zhaoran Wang

**Affiliations:** 1 School of Physical Education, Xihua University, Chengdu, China; 2 Department of Physical Education, Chengdu Textile College, Chengdu, China; 3 Department of Physical Education, Yuncheng University, Yuncheng, China; 4 College of Physical Education, Changwon National University, Changwon, Republic of Korea; 5 School of Physical Education, Qingdao University, Qingdao, China

**Keywords:** attention regulation, meditation, self-control, short, sports

## Abstract

**Background:**

Mindfulness-based interventions (MBIs) are psychological skills training approaches that are well recognized for enhancing psychological factors that influence sports performance. However, some factors, such as time commitment, cost, and accessibility required for traditional MBIs, can be a barrier to regular practice. As a result, brief MBIs with a duration of ≤ 30 min have gained popularity. This systematic review aims to explore the effect of brief MBIs on short sport-related performance.

**Method:**

A comprehensive search was conducted in five databases, including Web of Science, Scopus, PubMed, China National Knowledge Infrastructure, and EBSCOhost, for articles published up to 14 November 2025. The search utilized a combination of keywords related to brief MBIs and sport-related performance. The methodological quality of included studies was evaluated using the Cochrane Risk of Bias 2 (RoB 2) tool.

**Results:**

Ten randomized controlled trials met the inclusion criteria. Overall, brief MBIs demonstrated beneficial effects on several short sport-related performance outcomes, particularly precision-based motor skills. Significant improvements were observed in golf putting, hand grip strength, basketball tactical performance, and in some cases basketball free-throw shooting and soccer penalty kicks. However, results for basketball free-throw accuracy were mixed, and no significant effects were found for muscular endurance. The interventions varied in type, duration, and delivery mode, with most employing 4–30 min of audio-guided mindfulness practices. Risk of bias across studies was generally low, though inadequate reporting of randomization procedures was the most common concern.

**Conclusion:**

Brief MBIs lasting 30 min or less may induce short-term enhancements in certain domains of sport-related performance, particularly in tasks requiring attentional stability, fine motor control, and decision-making. However, these effects appear to be acute in nature, evidence remains inconsistent across sport types, and conclusions are limited by the small number of available studies. Future research should adopt standardized mindfulness assessments, include a broader range of physical performance indicators and relevant physiological measures to better elucidate the mechanisms underlying short-term brief MBIs effects in real competitive environments.

**Systematic review registration:**

https://inplasy.com/, INPLASY2022120086.

## Introduction

In sports, various psychological interventions have been developed to enhance athletic performance. One widely used approach is psychological skills training, which incorporates cognitive behavioral techniques such as goal setting, mental rehearsal, and arousal control (1–3). Another form of mental training that has gained increasing attention is mindfulness. Mindfulness is commonly defined as the purposeful, non-judgmental attention to the unfolding of present-moment experiences (4). Mindfulness encompasses therapeutic concepts such as acceptance, detachment, and compassion (5–7). Mindfulness-based interventions (MBIs) have been used to alleviate symptoms of depression, burnout, mental fatigue, and anxiety among athletes (8–10). The use of mindfulness interventions in sports originated in the early 1980s (11), and since then, it has gained popularity in the field of sports psychology. Mindfulness meditation has been shown to improve several psychological processes that influence sport performance, including attentional control, emotional regulation, stress reduction, and the ability to remain task-focused under pressure (12–14). These psychological mechanisms are central to athletes’ capacity to maintain performance consistency when facing competitive stressors (15, 16). However, although mindfulness training has well-documented psychological benefits, its effects on physical or physiological components of athletic performance, such as muscular strength, cardiorespiratory endurance, or neuromuscular output, remain far less established in the scientific literature (17, 18). This gap highlights the need for further research examining how mindfulness may influence both mental and physical determinants of sport performance.

Sport-related performance refers to the athletic performance that can impact the outcome of a sporting event, including cardiorespiratory endurance, muscular endurance, strength, motor skills, and decision-making abilities (19–21). It is influenced by both physical and psychological factors and can be affected by various stressors (22, 23), such as game pressure (24) or mental fatigue (25). Research has shown that mental fatigue can have a negative impact on basketball players’ performance (26). The reinvestment theory provides insight into this mechanism by suggesting that not all types of present-moment attention are beneficial for athletic performance (27, 28). Excessive self-focused attention and task-irrelevant distraction caused by performance evaluation can impair athletic performance (29). Athletes may become mentally preoccupied and lose focus on the task at hand when their expectations for the upcoming game and their evaluation of their current performance are not aligned (29).

Studies have shown that MBIs can counteract the negative effects of reinvestment on sport-related performance by helping individuals accept self-expectations and self-evaluations (30–33). The mindfulness model and de-automatization suggest that mindfulness can initiate subsequent mental processes that enhance attention control and self-regulation, both of which are essential for performance improvement (34–36).

There are several structured MBIs that have been developed in applied settings. The Mindfulness-Acceptance and Commitment approach (MAC), based on Acceptance and Commitment Therapy (ACT) (37), is one of the most popular mindfulness interventions in sports and typically involves multiple sessions delivered over several weeks, integrating acceptance, values clarification, and attentional training within sport-specific contexts (31, 38, 39). Another benefit of mindfulness intervention for the mental readiness of athletes is the Mindfulness Sports Performance Enhancement Program (MSPE) (40, 41), which adapts mindfulness practices to athletic settings through structured group-based training sessions (42–44). Similarly, the Mindfulness for Performance (MFP) program (45) and the Mindfulness Training Program (MTP) (46) incorporate mindfulness and acceptance principles into sport-specific training formats, often requiring repeated sessions and guided instruction. These programs highlight the growing variety of mindfulness approaches applied in sport settings and illustrate that mindfulness training can be tailored to different athletic demands.

Despite the benefits of MBIs for sport-related performance, there are barriers to their implementation. Traditional MBIs often require lengthy sessions delivered by trained therapists in specialized settings, making them less accessible and feasible for many athletes (31, 41). To address these limitations, an increasing number of studies have examined the effects of brief MBIs in sport contexts. Recent evidence shows that short-duration mindfulness protocols can enhance or restore sport-related performance across different tasks. For example, brief MBIs could improve or maintain basketball tactical performance after mental fatigue (47), enhance attentional control during penalty kicks in football (48), and counteract ego-depletion effects on motor skill performance under pressure (49). Brief MBIs focus on a single essential element of mindfulness, such as screening, body scan, or breath exercises (50). According to Howarth et al., brief MBIs are also defined by their duration, consisting of sessions of 30 min or less, with a total volume not exceeding 100 min per week and delivered for no more than 4 weeks. Compared to traditional MBIs that are suitable for off-season or periods when athletes can commit more time to mental training (51), brief MBIs can lead to immediate, short-term benefits such as reduced stress, improved focus, and enhanced mood (50, 52). Brief MBIs are useful for quick stress relief and improving immediate performance, but may not provide the deep, long-lasting changes in behavior and cognition seen with more extensive programs (53). Although Howarth et al. (50) do not focus specifically on athletes, brief interventions may be particularly valuable in sport contexts because athletes often face demanding training schedules, travel commitments, and limited recovery time, making it difficult to engage in longer mindfulness programs. As a result, brief MBIs offer a practical and time-efficient alternative for supporting athletes’ psychological readiness and performance during periods when extended mental training is not feasible (47, 54). Despite the growing interest and research on brief MBIs in sports psychology, there remains a fragmented understanding of their overall efficacy and mechanisms of action (48, 49). This review aims to bridge this gap by synthesizing findings from diverse studies, thus providing a more cohesive and comprehensive understanding of how brief MBIs may influence sport-related performance. By systematically reviewing the literature, this study attempts to identify the most effective brief MBIs techniques and their practical applications, thereby serving as a valuable resource for optimizing training programs and mental conditioning practices.

## Method

### Protocol and registration

This systematic review followed the Preferred Reporting Items for Systematic Reviews and Meta-Analyses (PRISMA) guidelines (55), and the completed PRISMA checklist is provided in the Supplementary materials. This title was registered on the Platform of Registered Systematic Review and Meta-analysis Protocols (INPLASY2022120086).

### Eligibility criteria

The inclusion criteria followed the PICOS (population, intervention, comparison, outcome, and study designs) criteria (Table 1). Articles were required to fulfil the following criteria:

1. Participants are mentally and physically healthy, with no restrictions on age, gender, ethnicity, or playing level.
2. In the experimental group, eligible studies implemented the brief MBIs, operationalized as a session lasting no more than 30 min, in line with our focus on the acute or immediate effects of mindfulness on sport-related performance (50).
3. MBIs were defined according to the established mindfulness frameworks, such as Mindfulness-Based Stress Reduction (MBSR), Mindfulness-Based Cognitive Therapy (MBCT), MAC or MSPE (38, 44, 56, 57). Other practices, such as yoga or Qigong, incorporate mindful components but are not classified as MBIs were not included. Intervention was required to consist solely of brief mindfulness-based practices. Studies in which mindfulness was delivered in combination with other components, such as nutritional supplements or physical treatments, were excluded to avoid confounding effects.
4. Control groups did not receive brief MBIs.
5. Outcome measures are sport-related performance (e.g., sprinting, throwing, jumping).
6. Only randomized controlled trials (RCT) were included.
7. A full-text article published in English or Chinese.

**Table 1:** Inclusion criteria.

Items	Detailed inclusion criteria
Population	Healthy, regardless of population characteristics, such as age, gender, ethnicity, and playing level
Intervention	Brief MBIs
Comparison	Without brief MBIs
Outcome	Encompassed the effects of brief MBIs on sport-related performance
Study designs	RCT

MBIs, mindfulness-based interventions; RCT, randomized controlled trials.

The exclusion criteria were as follows:

1. Reviews.
2. Used long-term mindfulness training intervention, defined as interventions involving sessions exceeding 30 min in duration or delivered across multiple sessions.
3. Unpublished.

### Information sources and search strategy

The initial search was conducted on 13 October 2024, followed by an updated search on 14 November 2025 to ensure inclusion of the most recent publications. Both searches used the same databases (Web of Science, Scopus, PubMed, CNKI, and EBSCOhost). The search terms were (brief OR short OR abbreviated OR minute OR “short term”) AND (Mindful* OR meditat* OR Yoga) AND (sport* OR exercis* OR perform* OR skill* OR game* OR play* OR Athlet* OR train* OR motor OR capacity OR “physical activity”). The related reference lists in the included articles were screened by two authors (SC and YC). In addition to database searching, we conducted supplementary searches using Google Scholar and the reference lists of included studies to identify grey literature and relevant articles that may not have been captured in the initial database search. The complete search strategy of all the databases is summarized in Table 2.

**Table 2:** Number of hits for the complete search strategy for the databases.

Database	Complete search strategy	Hits
Web of Science	((TI = (brief OR short OR abbreviated OR minute OR “short term”)) AND TI = (Mindful* OR meditat* OR Yoga)) AND TI = (sport* OR exercis* OR perform* OR skill* OR game* OR play* OR Athlet* OR train* OR motor OR capacity OR “physical activity”)	216
	((AB = (brief OR short OR abbreviated OR minute OR “short term”)) AND AB = (Mindful* OR meditat* OR Yoga)) AND AB = (sport* OR exercis* OR perform* OR skill* OR game* OR play* OR Athlet* OR train* OR motor OR capacity OR “physical activity”)	3,044
Scopus	TITLE-ABS-KEY (brief OR short OR abbreviated OR minute OR “short term” AND mindful* OR meditat* OR yoga AND sport* OR exercis* OR perform* OR skill* OR game* OR play* OR athlet* OR train* OR motor OR capacity OR “physical activity”)	5,072
PubMed	((brief[Title/Abstract] OR short[Title/Abstract] OR abbreviated[Title/Abstract] OR minute[Title/Abstract] OR “short term”[Title/Abstract]) AND (Mindful*[Title/Abstract] OR meditat*[Title/Abstract] OR Yoga[Title/Abstract])) AND (sport*[Title/Abstract] OR exercis*[Title/Abstract] OR perform*[Title/Abstract] OR skill*[Title/Abstract] OR game*[Title/Abstract] OR play*[Title/Abstract] OR Athlet*[Title/Abstract] OR train*[Title/Abstract] OR motor[Title/Abstract] OR capacity[Title/Abstract] OR “physical activity”[Title/Abstract])	2,407
CNKI	TKA = (brief OR short OR abbreviated OR minute OR “short term”) AND TKA = (Mindful* OR meditat* OR Yoga) AND TKA = (sport* OR exercis* OR perform* OR skill* OR game* OR play* OR Athlet* OR train* OR motor OR capacity OR “physical activity”)	67
EBSCOhost (SPORTDiscus with Full Text)	AB (brief OR short OR abbreviated OR minute OR “short term”) AND AB (Mindful* OR meditat* OR Yoga) AND AB (sport* OR exercis* OR perform* OR skill* OR game* OR play* OR Athlet* OR train* OR motor OR capacity OR “physical activity”)	397
	TI (brief OR short OR abbreviated OR minute OR “short term”) AND TI (Mindful* OR meditat* OR Yoga) AND TI (sport* OR exercis* OR perform* OR skill* OR game* OR play* OR Athlet* OR train* OR motor OR capacity OR “physical activity”)	24

### Study selection

Duplicate studies were removed using Endnote software (X20, Thomson Reuters, New York City, NY, United States). Two authors (SC and JL) independently screened the retrieved articles based on the search strategy and assessed the titles/abstracts for eligibility. The third author (ZW) then independently reviewed the selected articles according to the PICOS criteria. Any discrepancies were resolved through discussion among all authors. The agreement between raters was determined by Kappa statistic using SPSS (58).

### Data extraction

Data were extracted from the selected articles by two authors (SC and YC). The following information was collected: (1) population characteristics (e.g., age, gender); (2) mindfulness experience, (3) Interventions; (4) duration of interventions; (5) comparisons; (6) mindfulness measurement; (7) outcomes. The extracted data were checked by another author (JL) to ensure accuracy.

### Risk of bias assessment

The methodological quality of the randomized controlled trials was evaluated by two authors (SC and JL) using the Cochrane Risk of Bias 2 (RoB 2) tool, which is specifically recommended for evaluating the risk of bias in randomized trials (59).

### Data synthesis

Due to the heterogeneity and variations of interventions, comparisons, and outcome measures used in the included studies, conducting a formal meta-analysis, which aims to combine and summarize the results of multiple studies to arrive at a more precise estimate of the effect, is not feasible because the pooling of such diverse data could lead to misleading or non-generalizable conclusions (60). Instead, a narrative review was performed because a qualitative synthesis is more appropriate to highlight the range of findings and contextual differences among the studies. The characteristics and effectiveness of the included articles were described in the text and tables following the guidance from the Centre for Reviews and Dissemination (61).

## Results

### Study selection

**Figure 1:**
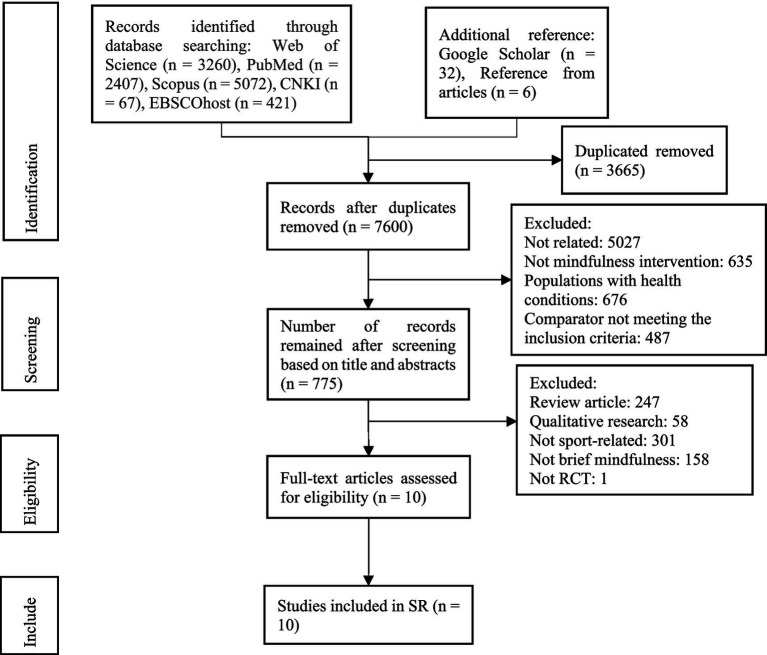
Systematic review search and screening procedure.

The literature screened 11,265 studies. After removing duplicates, 7,600 studies were reviewed by title and abstracts. Then, 670 full-length articles remained, and ten met the inclusion criteria (Figure 1). Studies that appeared to meet the inclusion criteria but were excluded after full-text screening, along with explicit reasons for exclusion, are presented in Supplementary Table S1. Inter-rater agreement during study selection was assessed using the Kappa statistic. The two reviewers initially achieved substantial agreement (*κ* = 0.81) during the title–abstract screening stage. Discrepancies were discussed and resolved through consensus, and the final agreement at the full-text screening stage reached perfect agreement (κ = 1.00).

### Effect of brief MBIs on short-term performance outcomes

The data extracted from the selected articles (Tables 3, 4) indicated that five studies examined the effects of brief MBIs on basketball performance. Three studies reported significant improvements in free-throw accuracy (49, 62) or tactical performance (47), whereas two studies found no statistically significant effects on free-throw shooting (54, 63). In addition, two studies assessed brief MBIs effects on soccer penalty kicks, with one showing a significant improvement (49) and the other reporting no effect (48). Finally, one study investigated golf-putting performance and demonstrated a significant enhancement following brief MBIs (64). On the other hand, two studies investigated the brief MBIs on muscular strength using the assessment tool of hand grip (65, 66), and the results showed that brief MBIs significantly increased sustained hand grip performance. One study investigated the brief MBIs on muscular endurance using the assessment tool of plank exercise, and there was no significant improvement (67).

**Table 3:** Overview of brief mindfulness-based intervention on sport-related performance.

Study	Population characteristics	Interventions	Experience	Comparisons	Measurement	Outcomes
Yusainy and Lawrence (66)	58 FM, 52 M; A = 19.5 ± 2.0 years; TL: recreationally active	Brief MBIs under ED	3 participants with ME	Listening excerpts and composing words	NR	Hand grip ↑ (*p* = 0.03) in EG vs. CG
Perry et al. (64)	32 FM, 33 M; A = 18.7 ± 0.8; TL: trained	Brief MBIs under pressure	EG: 25 participants with ME CG: 25 participants with ME	Sit quietly and read magazines	FFMQ	Golf putting task performance ↑ (*p* < 0.01, ES = 0.45) in EG vs. CG
Wang et al. (65)	46 FM, 14 M; A = 21.3 ± 1.0 years; TL: recreationally active	Brief MBIs under ED	All participants without ME	Sit quietly	NR	Hand grip ↑ (*p* = 0.027) in EG vs. CG
Stocker et al. (67)	18 FM, 16 M; A = 20.9 ± 1.3 years; TL: trained	Brief MBIs under ED	All participants without ME	Listening to audiobooks	TMS	Plank exercise ↔ (*p* = 0.36, ES = 0.03) in EG vs. CG
Shaabani et al. (62)	72 M; A = 28.6 ± 4.0 years; H = 193.0 ± 7.5 cm; BMI: 20.6 ± 2.0 TL: highly trained	Brief MBIs under ED	All participants without ME	Listening to an audio story	TMS	Basketball free-throw ↑ (*p* = 0.05, ES = 0.06) in EG vs. CG
Wolch et al. (63)	32 M; A = 21.2 ± 2.01 years; TL: trained	Brief MBIs under pressure	EG: 6 participants with ME CG: 6 participants with ME	Audio-recording on basketball history	TMS	Basketball free-throw ↔ (*p* = 0.28, ES = 0.04) in EG vs. CG
Chen et al. (54)	32 M; A = 19.8 ± 1.1 years; TL: trained	Brief MBIs under pressure	NR	Audio lectures or reports related to basketball	TMS	Basketball free-throw ↔ (p = 0.28) in EG vs. CG
Cao et al. (47)	54 M; A = 21.0 ± 1.1 years; H = 1.83 ± 4.56 cm; BM = 69.8 ± 6.6 kg; TL: trained	Brief MBIs under ED	All participants without ME	Reading basketball magazines	FFMQ	Basketball tactical performance ↑ (*p* < 0.05) in EG vs. CG;
Wagner and Wieczorek (49)	EG1: 7 FM, 11 M; A = 23.9 ± 3.1 years; EG2: 16 M; A = 23.1 ± 2.4 years; TL: recreationally active	Brief MBIs under ED	NR	Resting	NR	Basketball free-throw ↑ (p < 0.05, ES = 0.416) and penalty kicks ↑ (*p* < 0.001, η^2^ = 0.707) in EG vs. CG
Wu and Ai (48)	EG1: 40 M; A = 20.7 ± 2.4 years; EG2: 40 M; A = 22.1 ± 2.7; TL: trained	Brief MBIs under pressure	All participants without ME	Sit quietly	NR	Penalty kicks ↔ (*p* = 0.062, ES = 0.01) in EG vs. CG

A, age; C, control; FM, female; H, height; BM, body mass; BMI, body mass index; M, male; TL, training level; ME, mindfulness experience; NR, not reported; TMS, The Toronto mindfulness scale; FFMQ, five facet mindfulness questionnaire; EG, experimental group; ED, ego-depletion; CG, control group; ↑, significantly positive effect; ↔, no effect; ES = effect size.

**Table 4:** Mindfulness intervention.

Study	Duration	Delivery	Type	Mindfulness content
Yusainy and Lawrence (66)	15 min	Audio	MBCT-based	Mindfulness of body and breath: mindful breathing, body-scan-like awareness, noticing wandering thoughts, returning attention non-judgmentally; emphasis on decentering
Perry et al. (64)	30 min	Instructor-guided	MAC-based	Introduction to mindfulness; Discussion of task-focused and self-focused attention; Applied examples using participants’ sport experiences; “Brief centering exercise” (present-moment attention to sensations); Identification of mindfulness strategies for putting performance.
Wang et al. (65)	5 min	Audio	NR	NR
Stocker et al. (67)	4 min	Audio	MBCT-based	Breathing meditation focusing on breath and bodily sensations, noticing distractions, and gently returning attention to the present moment.
Shaabani et al. (62)	15 min	Audio	MBCT-based	Focused breathing induction; Breathing-and-body mindfulness directing attention to breath and bodily sensations; Instructions to notice distractions and gently return attention to the present moment.
Wolch et al. (63)	15 min	Audio	MM	Sitting meditation focusing on present-moment awareness of breath, bodily sensations, thoughts, and emotions; instructions to notice mind-wandering and gently return attention to breathing.
Chen et al. (54)	15 min	Audio	MM	Breath-focused meditation. Participants were instructed to focus on breathing sensations and gently return attention when distracted.
Cao et al. (47)	30 min	Audio	MAC	Centering exercise; Mindfulness of breathing; Body scan.
Wagner and Wieczorek (49)	15 min	Immersive VR	VR-based MM	Participants used flowborne, a VR breathing-based meditation game. Diaphragmatic breathing was trained through biofeedback: the VR controller detected abdominal movements, and each exhale produced real-time environmental changes to guide attention back to the breath.
Wu and Ai (48)	15 min	Audio	MM	Meditation using the “Yangqi” app, incorporating mindful breathing, body-scan elements, and non-judgmental present-moment awareness; participants were instructed to focus on breath and bodily sensations and gently return attention when distracted.

MBCT, Mindfulness-Based Cognitive Therapy; MAC, Mindfulness-Acceptance and Commitment; MM, Mindfulness meditation; VR, virtual reality; NR, not reported.

### Risk of bias assessment

**Figure 2:**
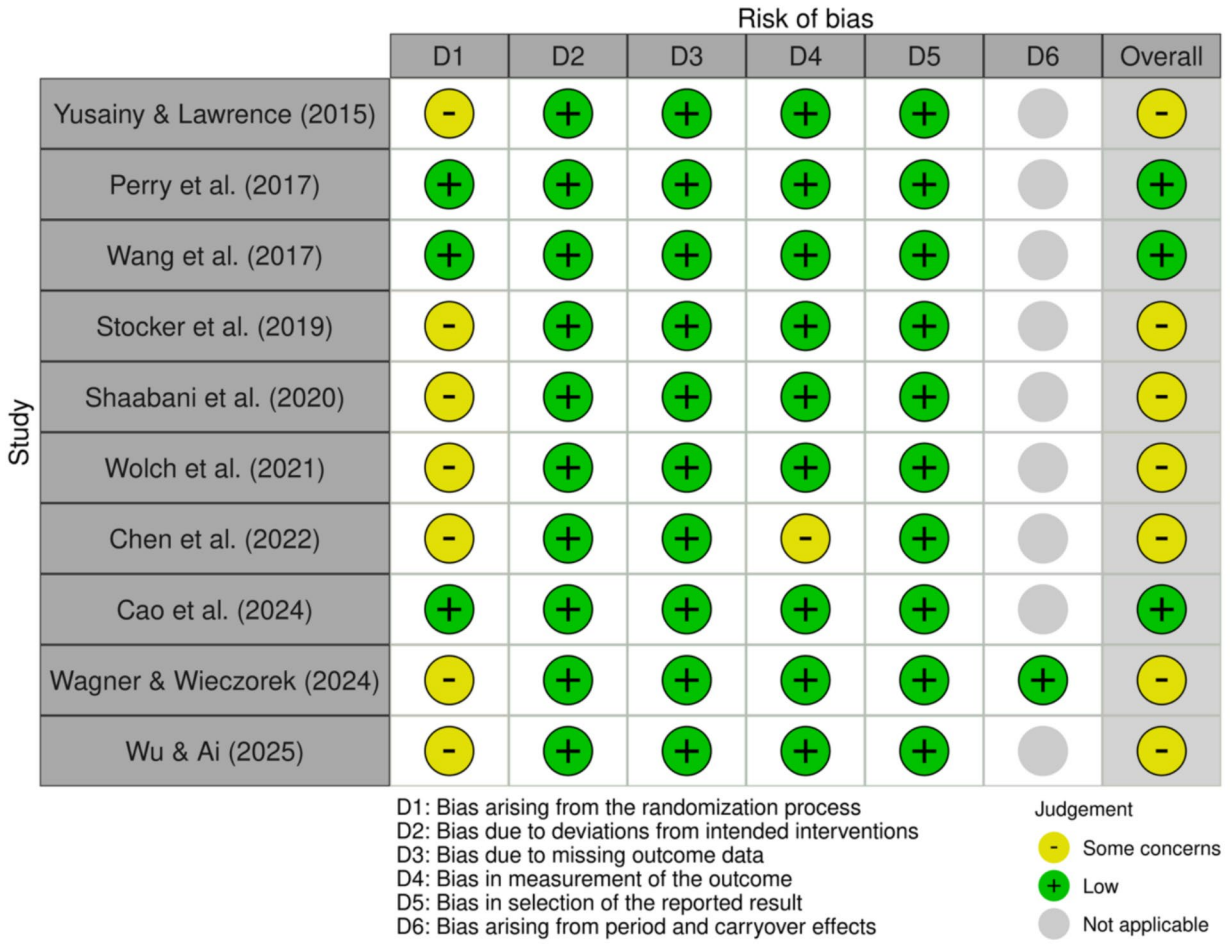
Risk of bias.

**Figure 3:**
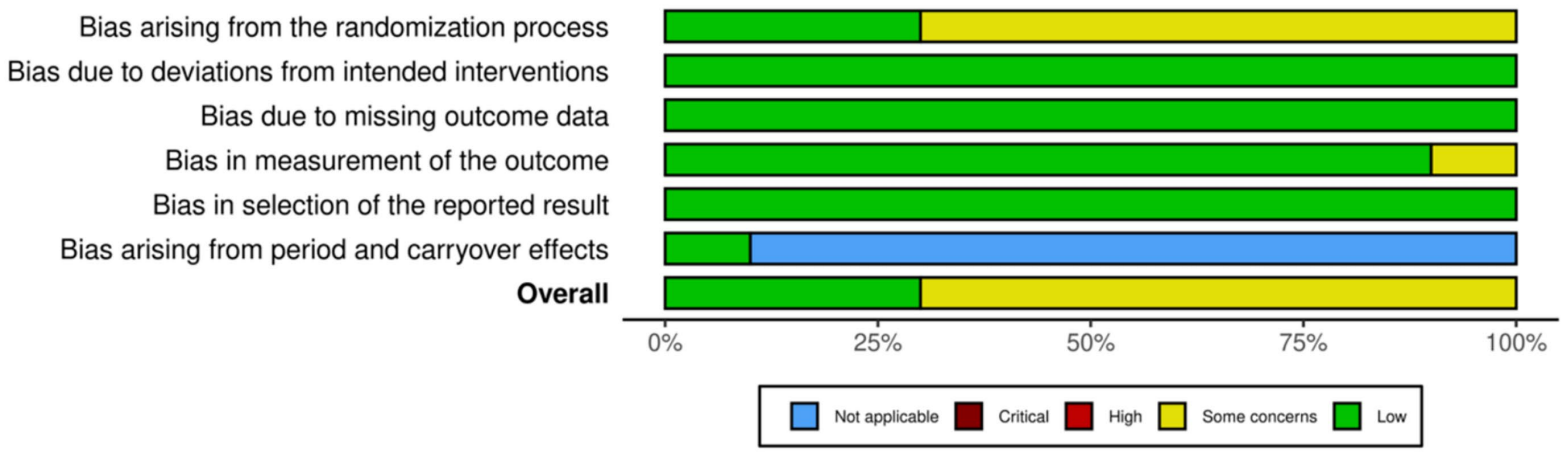
Risk of overall bias.

The risk of bias assessment indicated that most domains were predominantly judged as low risk of bias (Figures 2, 3). The main concern consistently arose from the randomization process, where several studies lacked sufficient information regarding allocation concealment. For the crossover RCT, all domains were rated low except Domain 1, which was judged as some concerns, leading to an overall judgment of some concerns. For the parallel RCT, most studies demonstrated low risk across Domains 2–5, while Domain 1 remained the primary source of concern in several studies. Finally, no study was judged as high risk in any domain.

## Discussion

Overall, the findings demonstrate that brief MBIs might have a positive effect on short-term performance domains, particularly precision-based motor skills (e.g., golf putting), tactical decision-making, and muscular strength, while showing inconsistent effects on basketball free-throw accuracy, soccer penalty kicks, and no measurable effect on muscular endurance.

The findings were mixed for basketball performance. Some studies reported significant improvements in free-throw performance following brief MBIs, while others found no effect. One possible explanation for this inconsistency relates to differences in age and training level of the participants. In Shaabani et al. (62), participants were approximately 29 years old and competed at a highly trained level, whereas participants in Wolch et al. (63), Chen et al. (54), and Wagner and Wieczorek (49) were younger (19–24 years old) and categorized as only trained players. First, highly trained players have likely mastered the fundamental physical skills and mechanics of free-throw shooting (68). Their training allows them to focus more on the mental aspects of performance (69), where mindfulness training can have a significant impact. In contrast, less trained players may still be working on the basic technical aspects of their game. As a result, their mental bandwidth might be consumed by physical execution (69), leaving less capacity to benefit from mindfulness training. Secondly, older players may benefit from the neuroplasticity associated with mindfulness (70), which can help refine neural pathways related to performance. Their extensive experience allows them to adapt these techniques more quickly and effectively. While neuroplasticity also benefits younger athletes, the immediate impact of brief MBIs might not be as pronounced if their neural pathways related to performance are still developing (70). Younger athletes are still in the process of stabilizing the motor patterns, attentional routines, and perceptual-cognitive strategies (71). These performance-related neural networks are not yet fully consolidated. Therefore, mindfulness may have fewer established pathways through which to exert rapid performance-enhancing effects (72). In contrast to free-throw shooting, one study reported significant improvements in basketball tactical performance after a 30-min MAC-based brief MBIs (47). Tactical tasks involve perceptual-cognitive processes such as decision-making, selective attention, and situational awareness (73). All these areas are known to benefit from mindfulness training. Since tactical performance was examined in only one study, the observed improvement cannot yet be generalized. More research is needed to confirm whether brief MBIs reliably enhances perceptual-cognitive performance in basketball.

Two studies assessed soccer penalty kick performance, with mixed results. Penalty kicks require integrating attentional focus, emotional regulation, and motor accuracy under pressure (74). Given the known benefits of mindfulness for attentional stability and anxiety reduction (75), improvements are theoretically plausible. The inconsistent findings may reflect variability in delivery mode (audio vs. VR) or training level of participants (recreationally active vs. trained). Golf putting showed the most consistent positive response to brief MBIs. Putting requires fine motor control, stable attentional focus, and low emotional interference (76). These domains are directly supported by mindfulness (75). Brief MBIs likely enhances performance in this context by improving concentration, reducing outcome-oriented rumination, and stabilizing attentional control (77, 78). In addition to enhancing general attentional stability, mindfulness also supports athletes in orienting attention toward goal-related cues (34, 36), a process that is central to sport performance (79). Mindfulness in sport involves not only noticing and accepting disruptive thoughts or emotions, but also the ability to rapidly refocus attention on task-relevant information such as movement cues, tactical options, or target-related features (79). This refocusing component may explain why brief MBIs appears particularly effective for precision-based tasks such as golf putting and, in some cases, basketball free-throws, where maintaining attention on task-critical cues is essential for performance consistency.

Two studies reported significant improvements in hand grip performance following brief MBIs. Hand grip is a common measure of muscular strength and requires sustained effort and tolerance of discomfort (80). The improvements may be explained by the role of mindfulness in enhancing interoceptive awareness and regulation of discomfort, reducing cognitive interference during effortful contractions, and replenishing self-control resources (81). However, because the included studies assessed only isometric handgrip performance, the extent to which these effects generalize to other components of muscular strength, such as maximal dynamic strength or explosive power, remains unclear.

Only one study evaluated muscular endurance using a plank test and found no significant effect. Muscular endurance relies primarily on physiological capacity and tolerance to sustained metabolic fatigue (82). Brief MBIs may not directly influence these physiological determinants, particularly when the duration of the mindfulness intervention is short (4 min). Furthermore, endurance tasks may involve prolonged physical discomfort, and a brief intervention may not be sufficient to meaningfully alter pain perception, motivation, or attentional drift across longer intervals (50, 83).

In this systematic review, four studies investigated the effects of brief MBIs on skill-related performance under pressure, while four studies explored its impact under the conditions of ego depletion. Perceived pressure was assessed through the measurement of state anxiety. The catastrophe model suggests that heightened cognitive anxiety experienced by athletes due to external factors, such as stress or choking, can lead to impaired performance (84). Additionally, anxiety has been shown to induce self-focus or distraction, resulting in decreased task-relevant attention (85). Mindfulness training has been found to directly regulate anxiety and improve performance (86, 87). Shaabani et al. (62) demonstrated that ego depletion induced by the Stroop task could decrease basketball free-throw performance. The self-control strength model indicated that ego depletion occurs when self-control strength is overexerted under high-pressure conditions (Stroop task), leading to a dominance of the bottom-up and stimulus-driven attentional system (88). This can disrupt attentional processes and result in decreased performance in selective attention tasks (89). However, in line with the “top-down” emotion regulation perspective (90), brief MBIs can inhibit irrelevant impulses and help maintain focus on the task at hand, leading to the recovery of attention and self-control strength, ultimately enhancing task-relevant performance (91, 92).

### Limitations

Several limitations of this review should be acknowledged. First, many selected articles were conducted in a laboratory setting (e.g., golf putting or basketball free-throw tasks) rather than in real competitive contexts, which may limit ecological validity. Second, several studies did not assess the mindfulness states using standardized and validated instruments, making it difficult to determine whether the intervention effectively altered mindfulness. In addition, the scope of performance outcomes assessed in the included studies was limited. Other important performance components, such as speed, muscular power, agility, and cardiorespiratory endurance, were absent. Finally, relevant Medical Subject Headings (MeSH) terms, such as athletic performance, mindfulness, and meditation, were not systematically applied in the database searches. To advance the present research question, future studies should test brief MBIs in actual game settings or as close to them as possible, incorporate standardized mindfulness assessments to clarify underlying mechanisms such as the FFMQ, Mindful Attention Awareness Scale (MAAS), or Mindfulness Inventory for Sport (MIS) (93), and evaluate a broader range of sport-related performance outcomes spanning both skill-based and physical performance domains.

## Conclusion

The findings of this systematic review suggest that brief MBIs potentially enhance certain aspects of sport-related performance, particularly short-term outcomes such as precision-based motor skills, tactical decision-making, and hand grip strength. However, the evidence remains inconsistent across performance domains, with mixed results for basketball free-throw accuracy, limited benefits for soccer penalty kicks, and no observed effects on muscular endurance. Given the small number of available trials and the narrow range of performance indicators examined, these conclusions should be interpreted with caution. Importantly, the current evidence does not allow us to determine whether brief mindfulness alone is sufficient to improve long-term sport performance. Brief MBIs appear to offer immediate or short-term attentional and self-regulatory benefits, but whether these effects translate into sustained performance changes over time remains unclear. More high-quality research, including studies conducted in real competitive environments and those assessing a wider range of physical and motor performance outcomes, is needed to clarify the potential and boundaries of brief MBIs in athletic settings.

### Implications

The findings of this review indicate that brief MBIs might provide short-term benefits for athletes by enhancing attentional stability, emotional regulation, and self-control. Coaches, sport psychologists, and practitioners may consider incorporating brief MBIs into pre-performance routines or training sessions when the goal is to support immediate readiness, reduce anxiety, or stabilize focus before skill execution. Because brief MBIs require minimal time, resources, and instruction, they offer a practical mental preparation strategy that can be integrated into busy training schedules without adding excessive cognitive load. However, given the limited and heterogeneous evidence base, brief MBIs should not yet be regarded as a standalone method for improving long-term physical or sport performance. Future studies are needed to examine whether repeated or combined mindfulness practices can produce sustained performance gains, to evaluate the effects of brief MBIs across a broader range of performance components (e.g., speed, power, agility), and to test these interventions in real competition contexts. Researchers should also employ standardized mindfulness assessments and explore potential moderators such as training level, prior mindfulness experience, and intervention type or duration.

## Data Availability

The original contributions presented in the study are included in the article/Supplementary material, further inquiries can be directed to the corresponding author.
