# Supplementary material for: Do brief mindfulness-based interventions improve sport-related performance? A systematic review
Source: Front Public Health. 2026 Feb 5;14:1702327. doi: 10.3389/fpubh.2026.1702327 (PMC12916373; doi:10.3389/fpubh.2026.1702327)
Supplement: Supplementary file 2 [file Data_Sheet_2.docx]

**Appendix B**

**Table S1: Studies excluded after full-text screening and reasons for exclusion**

| **N** | **Study** | **Reason for Exclusion** |
| --- | --- | --- |
| **1** | (Donohue et al., 2006) | Yoga intervention without an explicit mindfulness-based component (no formal mindfulness instruction or attentional training). |
| **2** | (Thangavel et al., 2014) | Long-term mindfulness intervention. |
| **3** | (Dobie et al., 2016) | Not sport-related. |
| **4** | (Zhu et al., 2020) | Brief mindfulness intervention combined with carbohydrate ingestion (co-intervention), confounding the isolated effects of mindfulness. |
| **5** | (Stapleton et al., 2020) | Outcomes were limited to EEG-based neurophysiological measures, with no assessment of sport-related performance. |
| **6** | (Aras et al., 2023) | Outcomes focused on physiological and cognitive recovery rather than sport-related performance. |

**Reference**

Aras, D., Onlu, A. S., Durmus, T., Cengiz, C., Guler, D., Guler, Y., Ugurlu, A., Aldhahi, M. I., & Gnln, M. (2023). A brief body scan mindfulness practice has no positive effect on the recovery of heart rate variability and cognitive tasks in female professional basketball players. *Frontiers in Psychology*, *14*, Article 1196066. <https://doi.org/10.3389/fpsyg.2023.1196066>

Dobie, A., Tucker, A., Ferrari, M., & Rogers, J. M. (2016). Preliminary evaluation of a brief mindfulness-based stress reduction intervention for mental health professionals [Review]. *Australasian Psychiatry*, *24*(1), 42-45. <https://doi.org/10.1177/1039856215618524>

Donohue, B., Miller, A., Beisecker, M., Houser, D., Valdez, R., Tiller, S., & Taymar, T. (2006). Effects of brief yoga exercises and motivational preparatory interventions in distance runners: Results of a controlled trial [Article]. *British Journal of Sports Medicine*, *40*(1), 60-63. <https://doi.org/10.1136/bjsm.2005.020024>

Stapleton, P., Dispenza, J., McGill, S., Sabot, D., Peach, M., & Raynor, D. (2020). Large effects of brief meditation intervention on EEG spectra in meditation novices [Article]. *IBRO Reports*, *9*, 290-301. <https://doi.org/10.1016/j.ibror.2020.10.006>

Thangavel, D., Gaur, G. S., Sharma, V. K., Bhavanani, A. B., Rajajeyakumar, M., & Syam Sunder, A. (2014). Effect of slow and fast pranayama training on handgrip strength and endurance in healthy volunteers [Article]. *Journal of Clinical and Diagnostic Research*, *8*(5). <https://doi.org/10.7860/JCDR/2014/7452.4390>

Zhu, Y., Sun, F., Li, C., & Chow, D. H. K. (2020). Acute effects of brief mindfulness intervention coupled with carbohydrate ingestion to re-energize soccer players: A randomized crossover trial [Article]. *International Journal of Environmental Research and Public Health*, *17*(23), 1-11, Article 9037. <https://doi.org/10.3390/ijerph17239037>
